# Morphologies of tungsten nanotendrils grown under helium exposure

**DOI:** 10.1038/srep42315

**Published:** 2017-02-14

**Authors:** Kun Wang, R. P. Doerner, M. J. Baldwin, F. W. Meyer, M. E. Bannister, Amith Darbal, Robert Stroud, Chad M. Parish

**Affiliations:** 1 Oak Ridge National Laboratory, Oak Ridge, TN, USA; 2 University of California-San Diego, La Jolla, CA, USA; 3 AppFive, Tempe, AZ, USA

## Abstract

Nanotendril “fuzz” will grow under He bombardment under tokamak-relevant conditions on tungsten plasma-facing materials in a magnetic fusion energy device. We have grown tungsten nanotendrils at low (50 eV) and high (12 keV) He bombardment energy, in the range 900–1000 °C, and characterized them using electron microscopy. Low energy tendrils are finer (~22 nm diameter) than high-energy tendrils (~176 nm diameter), and low-energy tendrils have a smoother surface than high-energy tendrils. Cavities were omnipresent and typically ~5–10 nm in size. Oxygen was present at tendril surfaces, but tendrils were all BCC tungsten metal. Electron diffraction measured tendril growth axes and grain boundary angle/axis pairs; no preferential growth axes or angle/axis pairs were observed, and low-energy fuzz grain boundaries tended to be high angle; high energy tendril grain boundaries were not observed. We speculate that the strong tendency to high-angle grain boundaries in the low-energy tendrils implies that as the tendrils twist or bend, strain must accumulate until nucleation of a grain boundary is favorable compared to further lattice rotation. The high-energy tendrils consisted of very large (>100 nm) grains compared to the tendril size, so the nature of the high energy irradiation must enable faster growth with less lattice rotation.

The environment of a magnetic confinement fusion device, such as a tokamak, will be one of the most brutal ever envisioned by material engineers. Specifically, the plasma-facing materials (PFMs) will be subjected to high temperature, high heat flux, high-flux irradiation by hard-spectrum neutrons, and bombardment by fuel (deuterium, tritium) and ash (helium) ions from the plasma. The divertor in the ITER tokamak, for instance, will use tungsten as the plasma-facing material, due to its low sputter yield and high melting temperature. Under ITER-like helium bombardment conditions of high He flux and high temperature, tungsten has been observed to grow a thick mat of “fuzz” nanotendrils, typically a few tens of nm in diameter and up to several microns in length^1^.

In order to better predict the service of ITER, or larger follow-up reactors, significant research effort on the growth and properties of these tungsten nanotendrils has been pursued (refs 2, 3, 4, 5, 6, 7, 8, 9, 10, 11, 12, 13, 14, 15, 16, 17, amongst many others). In particular, simulation methods such as molecular dynamics (MD) have indicated that trap mutation, the formation of a Frenkel pair as several helium atoms accumulate and force themselves onto a tungsten lattice site, serves to nucleate bubbles^6^. As a bubble accumulates more helium, it grows and will punch out tungsten interstitials or dislocation loops^18^. These punched interstitials or dislocation loops will be attracted to the surface and presumably can act as incipient nanofuzz. MD simulations have also shown that as fluence builds, the surface around the bubbles will distort and begin to form early fuzz-like structures^11^,^19^. Generally, however, the detailed mechanisms of growth, and the structure of the tendrils after high fluences and extended growth, are still generally unknown, although preliminary views of bubble and tendril growth have been provided by modelling^11^,^15^,^18^,^20^,^21^,^22^,^23^,^24^,^25^ and experiment^12^,^13^,^14^.

Therefore, we have studied the microstructure of nanotendril mats, and isolated nanotendrils, grown under very different conditions, in order to characterize and quantify the similarities and differences in the tendrils’ structures. This quantitative experimental information will help validate and inform models and theory approaches as they simulate the growth of progressively more mature nanotendril structures.

In these experiments, we have grown W-nanofuzz at low (50 eV) and high (12 keV) He impingement energies, and used transmission and scanning electron microscopies to compare the structures of the tendrils themselves.

## Results

**Figure 1:**
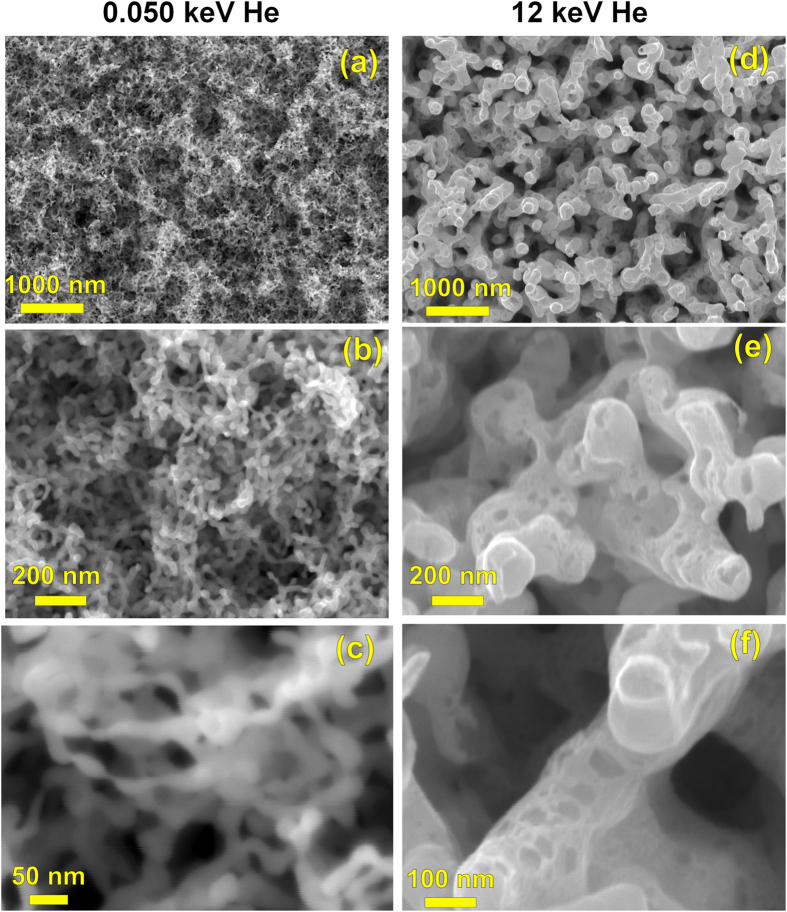
Plan-view SEM comparisons of the tendril structures produced by (**a**–**c**) low- and (**d**–**f**) high-energy He. SEM beam energies: (**a**–**d**,**f**) are 20 keV, (**c**) 5 keV.

The low-energy tendrils are far narrower than the high-energy tendrils, 22 ± 6 nm for the low-energy vs. 176 ± 15 nm for the high energy. Scanning electron microscopy (SEM) shows these gross differences in the growth morphologies between the low-energy (PISCES, Fig. 1a–c) and high-energy (MIRF, Fig. 1d–f) tendril samples. The surfaces of the low-energy tendrils (Fig. 1b,c) indicate the shape is generally round, with occasional constrictions, and either no surface pitting or unresolvably small (≪10 nm) surface pitting. The high-energy tendrils, on the other hand, are blockier, more uniform in thickness, and contain large numbers of surface depressions. The 50 eV tendril mat is ~1 μm deep, and the 12 keV tendril mat ~0.5 μm deep.

**Figure 2:**
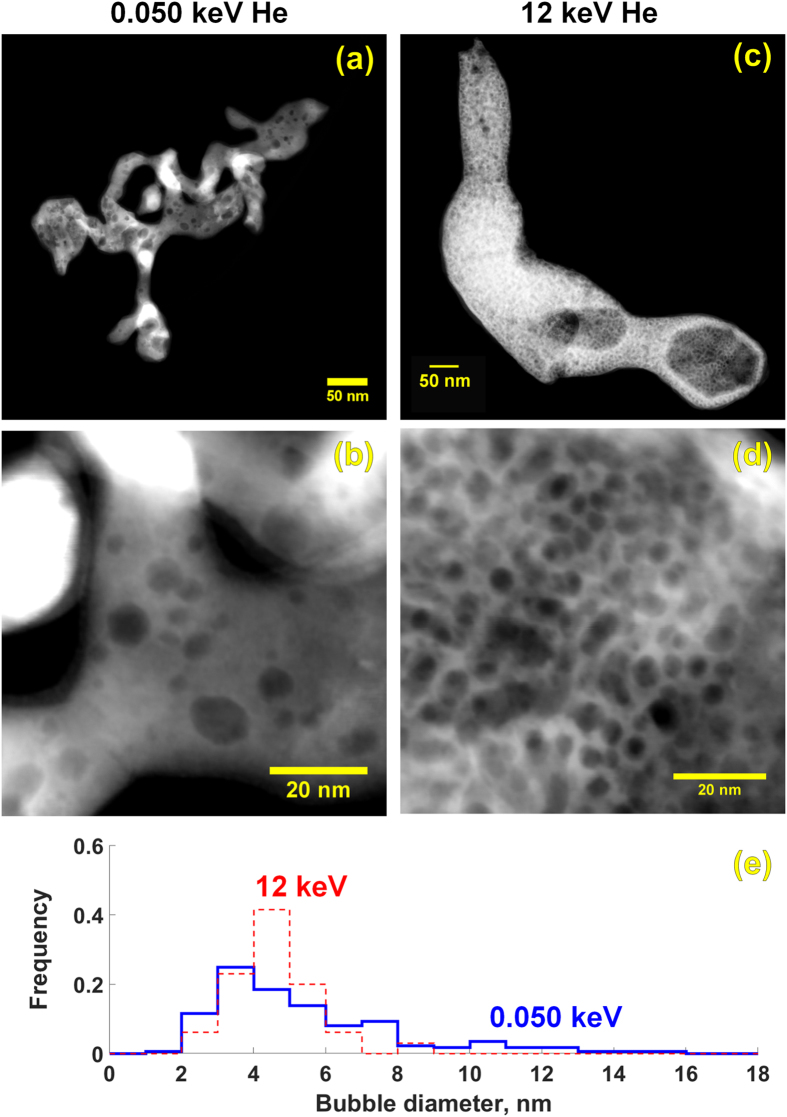
STEM-HAADF images of the different tendril structures. (**a**,**b**) low energy (**c**,**d**) high energy. (**e**) Histograms of bubbles sizes.

In STEM, the low-energy and high-energy tendrils are distinct. The low-energy tendrils are significantly smaller, and show clear internal bubble structures (Fig. 2a,b). The high-energy tendrils are thicker (resulting in less clear STEM imaging), and show larger bubbles and more complex bubble sub-structure (Fig. 2c,d). Bubble sizes in the 12 keV tendrils are difficult to measure, because the thick regions of the tendril are not sufficiently electron transparent, but bubble sizes in the thinner regions of the 12 keV tendrils, and throughout the 50 eV tendrils, are measured and quantified, Fig. 2e. The 12 keV tendrils show a smaller mean than the 50 eV tendrils, 4.1 ± 1.2 nm for the 12 keV and 5.1 ± 3.5 for the 50 eV.

**Figure 3:**
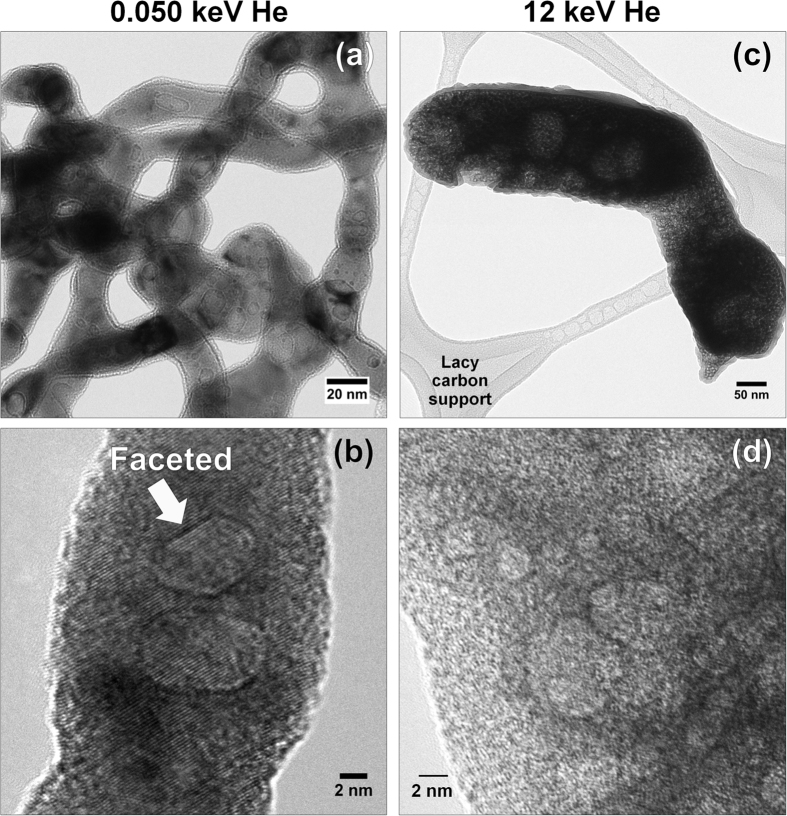
HRTEM images of the tendrils. (**a**,**b**) Low energy, showing faceted and rounded bubbles. (**c**,**d**) High-energy.

High-resolution TEM (HRTEM) of the tendrils shows the lattice planes (Fig. 3), and also shows clear faceting of some of the bubbles. This might indicate that in some of the bubbles, the internal gas pressure is relatively low, allowing low-surface-energy planes to form rather than minimizing the surface area of the bubbles as would be expected from highly overpressurized bubbles. Additionally, an amorphous edge is seen on the tendrils. This is likely a combination of thin native surface oxide (see below) and possibly some carbon contamination arising from residual organics from the specimen preparation procedure.

**Figure 4:**
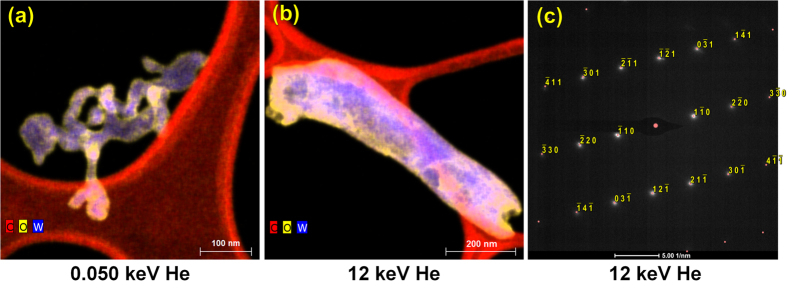
(**a**,**b**) X-ray maps showing W, C, and O for the two conditions. All maps have a 5 × 5-pixel kernel smoothing applied. (**c**) Selected area electron diffraction pattern from a different high-energy tendril, superimposed with calculated <113> diffraction pattern.

The high-energy tendrils give a mushy appearance (e.g., Fig. 3c,d), compared to the smoother low-energy tendrils. To confirm that this is a metallic tungsten structure, and not oxide, X-ray mapping and electron diffraction were performed. In the low-energy (Fig. 4a) and high-energy (Fig. 4b) structures, thin oxide layers (O = yellow) are seen on the edges of the W tendrils (=blue). The C (=red) in the maps are the lacy-carbon support films.

Although the O-rich layer appears slightly thicker in the high-energy tendril (Fig. 4b), this is probably an artifact of the larger-diameter tendril and the projection nature of (S)TEM data. Selected area electron diffraction of the high-energy tendril (Fig. 4c) indexes cleanly as W metal (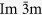) with no spots consistent with the complex WO_2_ or WO_3_ unit cells, indicating the oxide volume fraction is very small (and probably amorphous).

**Figure 5:**
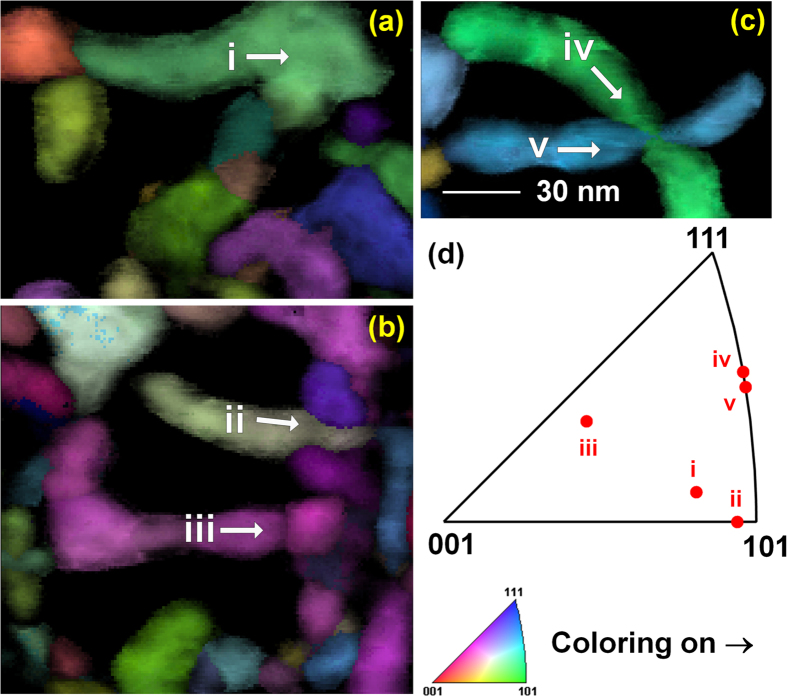
50 eV sample: (**a**–**c**) Crystallographic mapping images, with the inverse pole figure coloration projected on the horizontal (→) direction (Color, inset triangle; intensity, image quality). (**d**) shows the long-axis crystallographic directions of the five labelled grains. Superimposed white arrows indicate the approximate specimen directions of the long axes marked in (**d**). (All figures to same scale).

**Figure 6:**
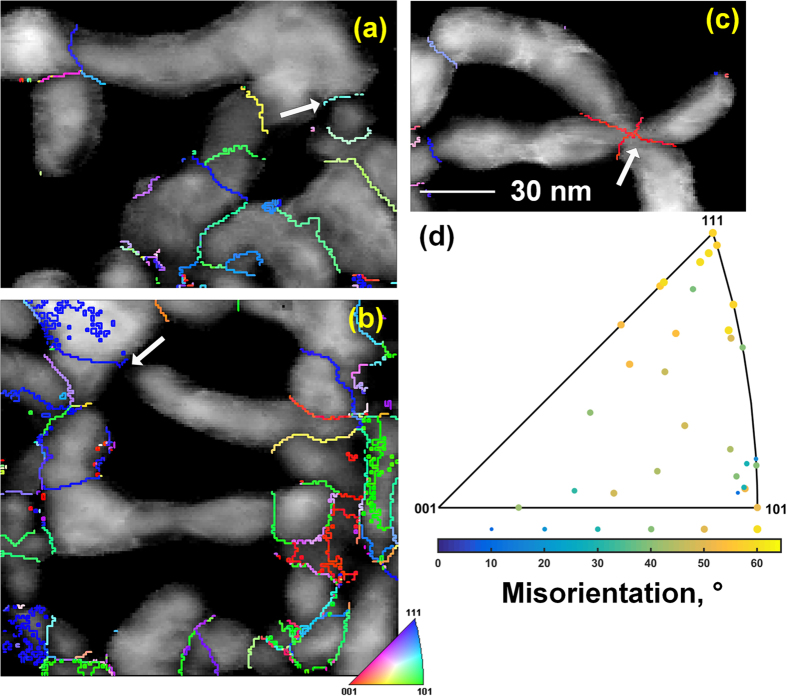
(**a**–**c**) PED image quality maps (grayscale) with superimposed grain boundary axes (colored by inset unit triangle). (**d**) Selected grain boundary axis/angle pairs, plotted onto the unit triangle as grain-boundary axis, marker size and color according to misorientation angle, as indicated by the key below the unit triangle. Arrows indicate examples of grain boundaries that are likely due to overlapping tendrils and not actual metallurgical grain boundaries. Densely speckled or checkerboard-like grain boundaries are pseudosymmetric misindexing. (All figures to same scale).

In the low-energy 50 eV tendrils, there does not appear to be a preferential growth direction of the grains, and the grain boundaries tend to be high angle. This is determined by precession electron diffraction (PED) crystallographic mapping (Figs 5 and 6). Grain crystallographic orientations are shown in Fig. 5a–c, colored by projecting the inset unit triangle onto the horizontal (→) direction; intensity is the image quality. In Fig. 5a–c, elongated grains are marked with Roman numerals i to v, and the long axes’ crystallographic orientations were measured and plotted in Fig. 5d. For the small number of grains (five) measured, no preferred orientation is detected. The slight color variations within the grains are indicative of slight differences in the measured orientation. However, the orientation measurements will be limited to about ±1° due to the way PED data is analyzed, so it would be difficult to measure small strains from these results. However, strain analysis methods are possible using similar techniques in TEM and may be worth attempting in the future.

Because the sample preparation may result in fuzz tendrils that were unassociated with each other falling together into mats on the TEM sample grids, it is more difficult to measure grain boundary axis/angle pairs. In Fig. 6a–c, several grain boundaries are arrowed. These are examples of grain boundaries we judge to likely be arising from overlapping tendrils and not actual growth boundaries of intimately joined grains. The arrow of Fig. 6c, for instance, is obviously marking unassociated tendrils that fell across each other. However, we conservatively found 31 grain boundaries that are very likely from actual growth-induced grains and measured the axis/angle pairs of these boundaries. Most of the boundary’s misorientation axes fall away from <001>, and there is no strongly preferred angle, although 50–60° is most common, Fig. 6d. Again, acknowledging a small sample size, there appears to be no strongly preferred axis, and the angles tend to be high. Few low-angle (<15°) grain boundaries were observed. Efforts are underway to obtain more comprehensive statistics; this is ongoing and will be reported elsewhere. In Fig. 6a–c, the image grayscales are colored by the image quality of the PED, and the grain boundaries are colored according to the grain boundary axis, as indicated by the inset colored unit triangle. A more detailed study, using transmission Kikuchi diffraction (tKD), of low energy tendrils is published elsewhere^26^, but agrees with these results.

**Figure 7:**
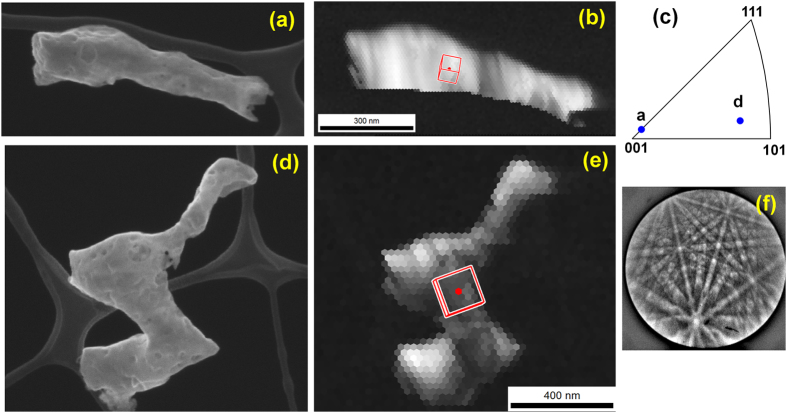
Transmission Kikuchi diffraction of the high-energy (12 keV) tendrils. (**a**,**d**) Secondary electron images. (**b**,**e**) Image quality maps with superimposed crystal unit cells. (**c**) The long axes of the tendrils. (**f**) A typical tKD pattern (from the scan in (**e**)).

Because the high-energy 12 keV tendrils were thicker, we obtained tKD patterns in SEM, rather than resorting to PED analysis in TEM. Examples of high-energy tendril tKD data are given in Fig. 7. The individual isolated tendrils tend to be single crystalline over several hundred nanometers, indicating a much larger grain size than the low-energy fuzz. Here, tKD is vital because the specimen thickness and dense bubbles make TEM grain-size analysis difficult. Although small in-grain rotations are present, the point-to-point misorientations are <15°. The grains in Fig. 7 are marked with the superimposed unit cell of the grain average orientation in red, and Fig. 7c shows the long axes of the two grains. A typical tungsten tKD pattern is shown in Fig. 7f.

## Discussion

The low-energy tendrils show a very complex structure, with the dominant features being nanocrystalline grains (~20–50 nm) along the tendril length axis, and ~4–10 nm sized bubbles within the grains and at the grain boundaries. The high-energy tendrils are dominated by a wider range of bubble sizes and surface depressions.

From a materials service point of view, the lower-energy case is more comparable to the expected flux onto a tokamak divertor under steady-state conditions, and tendrils growing with the types of morphologies and sizes observed here may appear under the steady-state conditions. In order to model or predict how such a layer, grown in-reactor, would behave, knowledge of the grain structure and bubble structures is necessary.

The mechanism of tendril growth is still not established, although the earliest stages have been modelled using MD^11^,^18^,^19^. The observations of the nanocrystalline nature of the tendrils^17^,^26^ indicates a continuous nucleation of new grains, or at least lattice rotation continuously to the point that grain walls can form, as the growth progresses. Whether new grains are nucleated at the tendril/substrate interface, or whether progressive rotation of the growing tendrils resulting in formation of boundaries when the growing orientation reaches sufficient magnitude, is still not determined.

High-energy helium bombardment, such as the 12 keV here, is less likely to be seen in-reactor. However, occasional off normal events (such as an ELM) may deliver keV-range He ions, and from a fundamental point of view, the differences in growth morphologies are indicative of differing mechanisms. This is important, because the mechanisms of growth of nanotendrils, at low or high energy, is not established in detail. The grain sizes of the high-energy tendrils are hundreds of nanometers (e.g., each of the two shown in Fig. 7 are single crystals), compared to a few dozen nanometers in the low energy tendrils. This may mean that the greater generation rate of point defects (due to knock-on) in the high-energy specimen allows for faster grain growth of those tendrils.

Although the growth rate of the high energy tendrils, in terms of μm growth/hour, is about 4× lower than the growth rate of the of the low-energy tendrils, the growth efficiency of the high-energy tendrils *in terms of μm growth/unit fluence* is about 50× higher than the low-energy tendrils. Therefore, the efficiency of tendril growth is far higher at high energy, and we wish to explain this. We speculate that the helium inventory in the high-energy case will be large, despite the lower absolute flux. First, the 12 keV He atoms are injected deep beneath the surface with a small (~25%) backscatter fraction^27^. Second, and more important, and the formation of Frenkel defects will guarantee a very high He capture percentage because the implanted He is expected rest in the vacancies or vacancy clusters produced in the displacement cascade due to its extremely low solubility in W. Conversely, about half of the low energy ions will be backscattered from the surface, and about half of the injected ions will diffuse to the surface and escape^6^. Further, the displacive formation of interstitials in the Frenklel pairs will also provide a further mechanism to drive tendril growth more rapidly; interstitials punched from growing bubbles is likely one of the root mechanisms of tendril growth^6^, and additional interstitials may enhance the growth efficiency. Therefore, we expect that the high capture fraction and formation of Frenkel pairs will result in the faster growth and thicker tendrils. The thin low-energy tendrils must bend or twist as they grow, which results in new grains nucleating: because the high-energy tendrils are much thicker than the low-energy tendrils, it will be more difficult for them to bend, so lattice rotations would be expected to be lower.

To summarize, we characterized the structures of high- and low-energy grown tungsten nanotendril “fuzz”. Both conditions form narrow grass-like mats of tendrils, with the low-energy fuzz being ≈8× narrower (22 ± 6 nm vs. 176 ± 15 nm). The high-energy fuzz has more surface topography. Transmission electron microscopy revealed the tendrils were nanocrystalline, with fine-scale cavities (almost certainly helium bubbles) within. The low-energy tendrils contained a unimodal size distribution of bubbles, and the high-energy tendrils contained a unimodal distribution of small bubbles and large surface depressions. It is possible that the thicker regions (unanalyzable with TEM) of the high-energy tendrils contain a different bubble size distribution than the thinner regions examined here, but we anticipate this difference to be small or negligible. Although surface oxide is present, the tendrils are unequivocally BCC tungsten. Although the mechanism of tendril growth is still unclear, we believe that comparing the low- and high-energy growth will allow further insights into this growth mechanism. Broadly speaking, these results indicate vastly different growth mechanisms under these two growth conditions. This implies engineering solutions to the amelioration of nanotendril growth, or surface degradation in general, will need to address multiple mechanisms of degradation.

## Methods

Two different systems were used to produce the tungsten tendrils. The first was the PISCES-A system at University of California-San Diego, described elsewhere^2^,^28^. The polycrystalline tungsten blank was polished to a mirror finish and exposed to He plasma at ~1200 K, 0.050 keV ion energy, ~10^23^ He^+^/m^2^sec, ~4 × 10^26^ He^+^/m^2^ total. Second, we used the ORNL Multicharged Ion Research Facility (MIRF), described elsewhere^29^,^30^. Samples were mirror-polished blanks and exposed at ~1300 K, 12 keV He ion energy, ~5 × 10^20^ He^+^/m^2^sec, ~4 × 10^24^ He^+^/m^2^ total^31^. Different tungsten stock was used for the two experiments. The low energy sample was press sintered from 99.95% pure tungsten powder obtained from Midwest Tungsten Service, Inc., (according to the specifications approved for ITER material selection^32^) while the high energy sample was cut from 0.7 mm thick hot rolled tungsten sheet assayed to be >99.99% pure W. Both samples were mechanically polished to a mirror finish and annealed prior to He exposure at 1000 °C for 1 hour. The tungsten grains in the low energy sample are elongated in the direction normal to the surface, while those on the high energy sample are elongated in a direction parallel to the surface and showed preferred <001> orientation near the surface. (It should be noted, however, that prior measurements of fuzz formation on a variety of different grades of tungsten showed little differences in the formation of the fuzzy layer^2^. A previous publication^33^ indicated only minor effects of starting grain size and dislocation density on tendril morphology, far smaller than the effects measured here, so we make the assumption that the minor differences in starting structure are negligible in comparison to the helium energy differences).

Samples were examined in plan-view using a JEOL JSM6500F field-emission scanning electron microscope (SEM) operated at 20 keV, or (for high magnification) an FEI Versa DualBeam operated at 5 keV, both at Oak Ridge National Laboratory, USA.

Scanning/transmission electron microscopy ((S)TEM) sample preparation was performed by physically removing the tendrils from the substrate, rather than using a more common TEM sample preparation method such as focused ion beam (FIB). TEM copper-mesh sample grids with lacy carbon or continuous carbon support films were gripped with tweezers, wet with a few drops of methanol, then a few drops of methanol were dripped onto the fuzzy tungsten surface, and then the TEM grid dragged across the tendril-coated tungsten surface by using the tweezers. This transferred tendrils to the TEM grid. This also avoided any sort of ion-beam damage as would be expected from FIB-based methods. TEM and STEM imaging and X-ray mapping was performed in an FEI Talos F200X system at Oak Ridge National Laboratory, USA^34^,^35^. Bubble sizes were measured using HAADF STEM, from edge to edge across the wide part of the bubble, using ImageJ (https://imagej.nih.gov/ij/) software to measure the widths across the dark features in the HAADF signal. Number densities and bubble spacings require knowledge of the tendril local thickness, which we plan to analyze in a follow-up publication.

Samples of the 50 eV tendrils were examined using precession electron diffraction (PED^36^) to produce crystallographic maps of the specimen at high resolution. This was performed at AppFive, Tempe, AZ, using a Philips CM200 (S)TEM and Nanomegas ASTAR system. PED used conditions of 200 keV, 2 nm step size, 0.4° precession angle, and 0.01 sec/pattern. Transmission Kikuchi diffraction (tKD^37^,^38^,^39^) was performed in the JEOL 6500 F, using an EDAX Hikari I electron backscatter diffraction (EBSD) camera, at 20–30 keV, 3–5 nA, with 6–7 mm working distance and a specimen tilt of negative 30° to the electron beam (where the tilt sense of conventional EBSD is considered positive).

## Additional Information

**How to cite this article**: Wang, K. *et al*. Morphologies of tungsten nanotendrils grown under helium exposure. *Sci. Rep.* **7**, 42315; doi: 10.1038/srep42315 (2017).

**Publisher's note:** Springer Nature remains neutral with regard to jurisdictional claims in published maps and institutional affiliations.

## References

[b1] Baldwin M. J. & Doerner R. P. Helium induced nanoscopic morphology on tungsten under fusion relevant plasma conditions. Nuclear Fusion 48, 035001, doi: 03500110.1088/0029-5515/48/3/035001 (2008).

[b2] Baldwin M. J. & Doerner R. P. Formation of helium induced nanostructure ‘fuzz’ on various tungsten grades. Journal of Nuclear Materials 404, 165–173, doi: 10.1016/j.jnucmat.2010.06.034 (2010).

[b3] Nishijima D., Baldwin M. J., Doerner R. P. & Yu J. H. Sputtering properties of tungsten ‘fuzzy’ surfaces. Journal of Nuclear Materials 415, S96–S99, doi: 10.1016/j.jnucmat.2010.12.017 (2011).

[b4] Wright G. M. . Tungsten nano-tendril growth in the Alcator C-Mod divertor. Nuclear Fusion 52, doi: 04200310.1088/0029-5515/52/4/042003 (2012).

[b5] Lasa A., Henriksson K. O. E. & Nordlund K. MD simulations of onset of tungsten fuzz formation under helium irradiation. Nuclear Instruments & Methods in Physics Research Section B-Beam Interactions with Materials and Atoms 303, 156–161, doi: 10.1016/j.nimb.2012.11.029 (2013).

[b6] Sefta F., Hammond K. D., Juslin N. & Wirth B. D. Tungsten surface evolution by helium bubble nucleation, growth and rupture. Nuclear Fusion 53, 073015 (2013).

[b7] Wong C. P. C. . In Fusion Engineering (SOFE), IEEE 25th Symposium on. 1–6 (2013).

[b8] Wright G. M. . Comparison of tungsten nano-tendrils grown in Alcator C-Mod and linear plasma devices. Journal of Nuclear Materials 438, S84–S89, doi: 10.1016/j.jnucmat.2013.01.013 (2013).

[b9] El-Atwani O. . Ultrafine tungsten as a plasma-facing component in fusion devices: effect of high flux, high fluence low energy helium irradiation. Nuclear Fusion 54, 083013 (2014).

[b10] Ueda Y. . Research status and issues of tungsten plasma facing materials for ITER and beyond. Fusion Engineering and Design 89, 901–906, doi: 10.1016/j.fusengdes.2014.02.078 (2014).

[b11] Ito A. . Molecular dynamics and Monte Carlo hybrid simulation for fuzzy tungsten nanostructure formation. Nuclear Fusion 55, 073013 (2015).

[b12] Takamura S. & Uesugi Y. Experimental identification for physical mechanism of fiber-form nanostructure growth on metal surfaces with helium plasma irradiation. Applied Surface Science 356, 888–897 (2015).

[b13] Woller K., Whyte D. & Wright G. Dynamic measurement of the helium concentration of evolving tungsten nanostructures using Elastic Recoil Detection during plasma exposure. Journal of Nuclear Materials 463, 289–293 (2015).

[b14] Fiflis P., Curreli D. & Ruzic D. Direct time-resolved observation of tungsten nanostructured growth due to helium plasma exposure. Nuclear Fusion 55, 033020 (2015).

[b15] Lasa A., Tähtinen S. & Nordlund K. Loop punching and bubble rupture causing surface roughening—a model for W fuzz growth. EPL (Europhysics Letters) 105, 25002 (2014).

[b16] Yang Q. . Nanostructured fuzz growth on tungsten under low-energy and high-flux He irradiation. Scientific Reports 5 (2015). 10.1038/srep10959 PMC4468520 26077598

[b17] De Temmerman G. . Nanostructuring of molybdenum and tungsten surfaces by low-energy helium ions. Journal of Vacuum Science & Technology A 30, 041306 (2012).

[b18] Wang J., Niu L.-L., Shu X. & Zhang Y. Stick-slip behavior identified in helium cluster growth in the subsurface of tungsten: effects of cluster depth. Journal of Physics: Condensed Matter 27, 395001 (2015). 26360187 10.1088/0953-8984/27/39/395001

[b19] Pentecoste L. . Low Energy and low fluence helium implantations in tungsten: Molecular dynamics simulations and experiments. Journal of Nuclear Materials 470, 44–54 (2016).

[b20] Krasheninnikov S. & Smirnov R. He cluster dynamics in fusion related plasma facing materials. Nuclear Fusion 55, 073005 (2015).

[b21] Smirnov R., Krasheninnikov S. & Guterl J. Atomistic modeling of growth and coalescence of helium nano-bubbles in tungsten. Journal of Nuclear Materials 463, 359–362 (2015).

[b22] Sandoval L., Perez D., Uberuaga B. P. & Voter A. F. Competing Kinetics and He Bubble Morphology in W. Physical Review Letters 114, 105502 (2015). 25815946 10.1103/PhysRevLett.114.105502

[b23] Perez D., Sandoval L., Uberuaga B. P. & Voter A. F. The thermodynamic and kinetic interactions of He interstitial clusters with bubbles in W. Journal of Applied Physics 119, 203301 (2016).

[b24] Krasheninnikov S. & Smirnov R. He cluster dynamics in W in the presence of cluster induced formation of He traps. Physica Scripta T167, 014021 (2016).

[b25] Hu L., Hammond K. D., Wirth B. D. & Maroudas D. Interactions of mobile helium clusters with surfaces and grain boundaries of plasma-exposed tungsten. Journal of Applied Physics 115, 173512, doi: doi: 10.1063/1.4874675 (2014).

[b26] Parish C. M., Wang K., Doerner R. P. & Baldwin M. J. Grain orientations and grain boundaries in tungsten nonotendril fuzz grown under divertor-like conditions. Scripta Materialia 127, 132–135, doi: 10.1016/j.scriptamat.2016.09.018 (2017).

[b27] Meyer F. . Flux threshold measurements of He-ion beam induced nanofuzz formation on hot tungsten surfaces. Physica Scripta T167, 014019 (2016).

[b28] Goebel D., Campbell G. & Conn R. Plasma surface interaction experimental facility (PISCES) for materials and edge physics studies. Journal of Nuclear Materials 121, 277–282 (1984).

[b29] Hijazi H. & Meyer F. W. A large-acceptance beam-deceleration module for retrofitting into ion-source beam lines. Review of Scientific Instruments 84, 033305, doi: 10.1063/1.4794740 (2013). 23556813

[b30] Meyer F. W. *et al. Recent activities at the ORNL multicharged ion research facility (MIRF*), http://accelconf.web.cern.ch/AccelConf/ECRIS2010/papers/mocobk04.pdf (2012).

[b31] Meyer F. W. . He-ion and self-atom induced damage and surface-morphology changes of a hot W target. Physica Scripta T159, 014029 (2014).

[b32] Material Specification for the Supply of Tungsten Bars for the ITER Divertor IDM Number: ITER_D_2×38PN -v. 1.0 (2010).

[b33] Wang K., Bannister M. E., Meyer F. W. & Parish C. M. Effect of starting microstructure on helium plasma-materials interaction in tungsten. Acta Materialia 124, 556–567 (2017).

[b34] Parish C. M. MT3FT-15OR0204122: Report on the acquisition and installation of FEI Talos F200X S/TEM. (Oak Ridge National Laboratory (ORNL), Oak Ridge, TN (United States). http://www.osti.gov/servlets/purl/1234344/ (2015).

[b35] Parish C. M. . LAMDA: Irradiated-Materials Microscopy at Oak Ridge National Laboratory. Microscopy and Microanalysis 21, 1003–1004 (2015).

[b36] Midgley P. A. & Eggeman A. S. Precession electron diffraction–a topical review. IUCrJ 2, 126–136 (2015). 10.1107/S2052252514022283 PMC4285886 25610633

[b37] Keller R. R. & Geiss R. H. Transmission EBSD from 10 nm domains in a scanning electron microscope. Journal of Microscopy 245, 245–251 (2012).

[b38] Trimby P. W. Orientation mapping of nanostructured materials using transmission Kikuchi diffraction in the scanning electron microscope. Ultramicroscopy 120, 16–24, doi: 10.1016/j.ultramic.2012.06.004 (2012). 22796555

[b39] Sneddon G. C., Trimby P. W. & Cairney J. M. Transmission Kikuchi diffraction in a scanning electron microscope: A review. Materials Science and Engineering: R: Reports 110, 1–12 (2016).

